# Pharmacokinetic Analysis of Weekly Versus Biweekly IgPro20 Dosing in Patients With Primary Immunodeficiency

**DOI:** 10.1002/cpdd.753

**Published:** 2019-12-08

**Authors:** Mikhail A. Rojavin, Hugo Chapdelaine, Michael A. Tortorici, Michaela Praus, Gautam Baheti, Ying Zhang, Jutta Hofmann, Roxane Labrosse, Renée Dicaire, Elie Haddad

**Affiliations:** ^1^ CSL Behring LLC King of Prussia Pennsylvania USA; ^2^ Department of Medicine Centre Hospitalier de l'Université de Montréal Université de Montréal Montreal Quebec Canada; ^3^ Institut de Recherches Cliniques de Montreal Montreal Quebec Canada; ^4^ CSL Behring GmbH Marburg Germany; ^5^ CSL Behring AG Bern Switzerland; ^6^ CHU Sainte‐Justine University of Montreal Montreal Quebec Canada; ^7^ Department of Pediatrics and Department of Microbiology Infectiology and Immunology University of Montreal Montreal Quebec Canada

**Keywords:** IgPro20, pharmacokinetic, primary immunodeficiency

## Abstract

Flexible dosing of IgPro20 (Hizentra®, CSL Behring, King of Prussia, Pennsylvania) maintains normal serum immunoglobulin G (IgG) levels in patients with primary immunodeficiencies (PID). Until now, clinical trials testing the pharmacokinetic (PK) characteristics of serum IgG of weekly and biweekly subcutaneous IgG therapy were not published. This is the first study assessing PK characteristics following weekly and biweekly IgPro20 in patients with PID. The PK study was conducted in 2 parts: weekly dosing (12 weeks) and biweekly dosing (up to 12 months). Serum IgG concentration‐time data were analyzed using noncompartmental methods to generate PK parameters. Fifteen patients provided PK samples for both dosing regimens. For weekly and biweekly regimens, mean doses per infusion were 109 and 213 mg/kg, respectively, and median t_max_ was 2.0 and 3.02 days, respectively. The mean C_trough_ values were similar in weekly and biweekly regimens (10.21 and 10.13 g/dL, respectively). The geometric mean ratios (GMRs) with 90% confidence intervals of biweekly to weekly C_max_ and C_trough_ were 1.10 (1.06–1.13) and 0.98 (0.95–1.01), respectively. The GMR of dAUC was 1.07 (1.03–1.10). This PK analysis demonstrated similar systemic IgG exposure after weekly and biweekly IgPro20 dosing with an equivalent monthly dose in patients with PID.

Primary immunodeficiencies (PID) are a heterogeneous group of disorders, most of which arise from intrinsic genetic defects leading to a dysfunctional immune system affecting antibody (Ab) production.^1^, ^2^ The standard treatment for PID with deficient Ab production is the use of immunoglobulin G (IgG) replacement therapy.^3^, ^4^, ^5^, ^6^, ^7^ Therapeutic IgG substitution can be administered either as intravenous (IVIG) or subcutaneous (SCIG) infusion. IVIG is the most common method of IgG administration in many countries; however, SCIG has emerged as an effective, convenient, and well‐tolerated alternative.^3^, ^5^, ^6^, ^8^

SCIG and IVIG have been shown to be equally effective; however, SCIG is reported to have lower rates of systemic adverse events with more stable serum IgG levels than IVIG.^9^, ^10^, ^11^, ^12^ IVIG results in an immediate steep rise in serum IgG level that declines with the redistribution of IgG into the extracellular space, giving rise to a potential “wear off” during the last week of the treatment interval.^6^ Alternatively, the frequent and low‐dosing schedule of SCIG provides a more stable serum IgG profile, similar to the IgG levels maintained in healthy individuals.^6^, ^13^ Unlike IVIG, SCIG does not require venous access, thereby offering greater convenience and better quality of life.^3^, ^4^, ^10^, ^14^, ^15^

Another key benefit of SCIG is the flexibility of dosing regimens, facilitating an individualized approach in dosing schedules for patients.^16^ This flexible dosing is supported by pharmacokinetic (PK) modeling and simulation, demonstrating that doses can be administered at varying intervals (daily to biweekly) with little impact on serum IgG levels.^16^, ^17^, ^18^ A study evaluating the PK characteristics of biweekly 16% SCIG dosing in 12 patients reported that this dosing regimen was well tolerated and resulted in the same serum IgG concentration as weekly infusions.^19^

Several SCIG formulations are available for the treatment of patients with PID, including varied IgG concentrations (10%, 16%, 20%) and hyaluronidase‐facilitated SCIG, which can further provide flexibility in dosing (up to 4 weeks between doses).^12^, ^20^, ^21^ IgPro20 (Hizentra®, CSL Behring, King of Prussia, Pennsylvania) was the first 20% liquid IgG formulation (with high purity, ≥98% IgG) approved globally for SCIG administration in patients with PID.^22^ The PK properties of IgPro20 in patients with PID are well established for weekly dosing^23^, ^24^, ^25^; however, PK data for biweekly dosing are not currently available.

In this study, a PK analysis was performed to evaluate the characteristics of weekly and biweekly SCIG IgPro20 administration in patients with PID for the same total dose.

## Methods

### Study Design

The study was approved by Centre Hospitalier Universitaire Sainte‐Justine Institutional Review Board (MP‐21‐2016‐1023) and took place at the Centre Hospitalier Universitaire Sainte‐Justine and Institut de Recherches Cliniques de Montreal. All the participants provided informed consent. This PK analysis was conducted in a subset of a population of a prospective, open‐label phase 4 study (NCT02711228) conducted in patients with PID. The PK substudy was conducted in 2 parts: part 1 (weekly dosing regimen) and part 2 (biweekly dosing regimen). All patients received stable IgPro20 doses for IgG replacement therapy prior to the start of the study. In part 1, patients continued their stable weekly IgPro20 regimen for 12 weeks. In part 2, patients received biweekly IgPro20 at twice their individual weekly dose received in part 1 and were observed for a period of up to 52 weeks. Patients from whom at least 1 postinfusion blood PK sample was collected and analyzed for 1 of the 2 study parts were included. For weekly dosing regimen, serum IgG concentration measurements were performed 6 weeks after the first dose. Patients completed at least 6 PK collection timepoints, with quantifiable concentrations in each part of the study, including a sample on day 8 for the weekly regimen and on day 15 for the biweekly regimen (Table 1). IgPro20 was administered subcutaneously using a SCIG60 Infusion Pump (EMED VersaRate Flow Rate Controller and EMED Soft‐Glide Multi‐Needed SUB‐Q Infusion Set). Infusions were most often administered in the abdomen and/or thighs. Serum IgG concentrations were measured at the local Clinical Laboratory Improvement Amendments‐certified core central laboratory using immunoturbidimetric test with ARCHITECT cSystems Analyzer (Abbott Diagnostics, Lake Forest, Illinois). Between‐run test‐retest reproducibility of the method coefficient of variance (CV) was ≤3.4%.

**Table 1:** PK Sampling Timepoints

Part 1 (Week 6)	
Day 1	Preinfusion (5–10 minutes before infusion)
Day 1	After end of infusion (5–10 minutes after infusion)
Day 2	24 ± 3 hours after start of infusion
Day 3	48 ± 3 hours after start of infusion
Day 4	72 ± 3 hours after start of infusion
Day 6	120 ± 3 hours after start of infusion
Day 8	168 ± 3 hours after start of infusion/5–10 minutes before next infusion

Part 2 (Week 12)	
Day 1	Preinfusion (5–10 minutes before infusion)
Day 1	After end of infusion (5–10 minutes after infusion)
Day 2	24 ± 3 hours after start of infusion
Day 3	48 ± 3 hours after start of infusion
Day 4	72 ± 3 hours after start of infusion
Day 6	120 ± 3 hours after start of infusion
Day 8	168 ± 3 hours after start of infusion
Day 15	5–10 minutes before next infusion

PK, pharmacokinetic.

### PK Parameters

A noncompartmental analysis (NCA) was performed using the serum IgG concentration values obtained for weekly and biweekly regimens using WinNonlin version 6.3 (Phoenix Build 6.3.0.395). The NCA was used to calculate the following PK parameters, which were compared between weekly and biweekly dosing regimens: area under the serum IgG concentration‐time curve from time zero through tau (AUC_0‐tau_; tau = 7 days [168 ± 3 hours] after start of infusion for weekly regimen and 14 days for biweekly regimen); dose‐adjusted AUC_0‐tau_ (dAUC, calculated as AUC_0‐tau_/actual dose); trough IgG concentration in serum collected before next infusion during a treatment regimen (C_trough_); maximum (C_max_) and minimum (C_min_) IgG concentration in serum during a single dosing interval; time to reach C_max_ (T_max_); clearance at steady state (CL_ss_, calculated as actual dose/AUC_0‐tau_).

### PK Statistical Analysis

Serum IgG concentrations and PK parameters were summarized by treatment regimen using descriptive statistics including the geometric mean and CV, in addition to the number of patients, the mean, standard deviation (SD), median, minimum, and maximum. The comparison of dAUC between the dosing regimens was based on geometric mean ratio (GMR) and corresponding 90% confidence interval (CI). In addition, the comparison of the non‐dose‐normalized C_max_, C_min_, and C_trough_ between the dosing regimens was also based on the GMR and corresponding 90%CI. For these analyses, values of dAUC, C_max_, C_min_, and C_trough_ were log‐transformed, and within‐patient differences were calculated. The mean of these differences (90%CI) was calculated. The back‐transformed mean and CI then constituted the GMR and its 90%CI limits.

## Results

### Patient Demographics and Disposition

Of 17 patients enrolled in the PK substudy, 15 patients provided samples for both weekly and biweekly dosing regimens. The major reasons for excluding patients from the PK population were: deviation of the planned dosing by more than ±10%, insufficient PK sample collection, and no participation in PK part 2 of the study. At the time of enrollment, 11 patients were diagnosed with common variable immune deficiency and one each with X‐linked agammaglobulinemia and X‐linked hyper‐IgM syndrome. Overall, the mean age of patients was 30.6 years, and 47% of patients were male (Table 2).

**Table 2:** Patient Demographic Characteristics

Number of patients	17
Age (years)	
Mean (SD)	30.6 (17.8)
Median (range)	19.0 (14–66)
Age category (years), n (%)	
≥12 and <16	3 (17.6)
≥16 and <18	4 (23.5)
≥18	10 (58.8)
Sex, n (%)	
Female	9 (52.9)
Male	8 (47.1)
Race, n (%)	
Caucasian	17 (100)
PID diagnosis, n (%)	
XLA	1 (5.9)
XHIM	1 (5.9)
Other	4 (23.5)
CVID	11 (64.7)
Baseline body weight (kg)	
Mean (SD)	71.4 (12.1)
Median (range)	72.5 (50.6–96)
Baseline BMI (kg/m^2^)	
Mean (SD)	25.3 (5.1)
Median (range)	24.2 (19.2–37)

BMI, body mass index; CVID, common variable immune deficiency; PID, primary immunodeficiency; PK, pharmacokinetic; SD, standard deviation; XHIM, X‐linked hyper‐IgM syndrome; XLA, X‐linked agammaglobulinemia.

### IgPro20 Infusions

As expected, the mean biweekly dose per infusion was approximately 2‐fold higher than the weekly dose. The mean total infusion volume for the biweekly dosing regimen was approximately double that for the weekly dosing regimen. The biweekly dosing regimen also had a slightly higher duration of infusion, mean number of injection sites per infusion, and volume per injection site than the weekly dosing regimen (Table 3).

**Table 3:** IgPro20 Infusion Parameters by Treatment Regimen

Mean (SD) Infusion Parameters, Unit	Weekly (n = 15)	Biweekly (n = 15)
Total infusion volume, mL	39.0 (8.3)	77.3 (16.7)
Dose per infusion, mg/kg bw	109 (22.8)	213 (41.1)
Infusion duration, min	49.2 (18)	66 (30)
Volume per injection site, mL	14.1 (4.1)	18.3 (4.0)
Number of injection sites	3.0 (1.1)	4.3 (0.6)

bw, body weight; min, minutes; SD, standard deviation.

### Serum IgG Parameters

**Figure 1:**
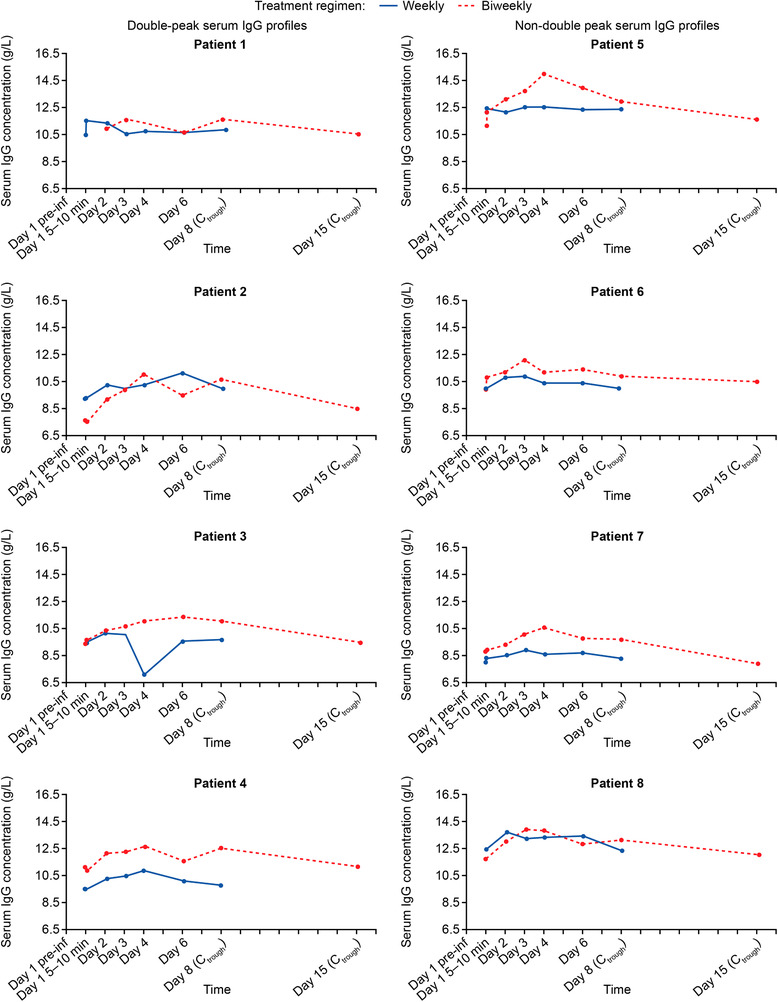
Individual serum IgG concentration‐time profiles. Serum IgG concentration over time for 8 patients is shown. Double‐peak profiles were observed in patients 1–4 and 8. Patients 1, 4, and 8 showed >5% change, Patients 2 and 3 showed >10% change. A threshold of 5% was conservatively selected as a true change indicator based on the reproducibility of the IgG assessment method. Preinfusion concentration, first PK sample of each PK sampling collection. C_trough_, trough IgG concentration in serum, collected before next infusion during a treatment regimen; IgG, immunoglobulin G; PK, pharmacokinetic.

**Figure 2:**
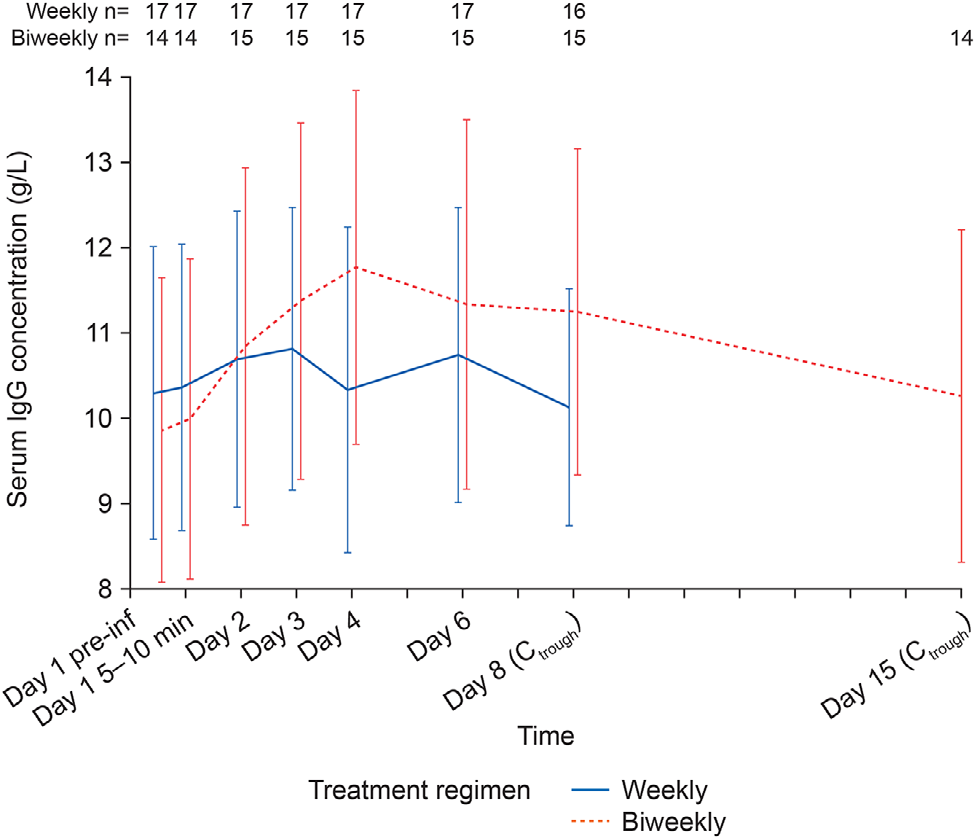
Average serum IgG concentration‐time profile. Mean (SD) serum IgG concentration over time is shown. Preinfusion concentration = first PK sample of each PK sampling collection. C_trough_, trough IgG concentration in serum, collected before next infusion during a treatment regimen; IgG, immunoglobulin G; PK, pharmacokinetic; SE, standard error.

Descriptive summaries of serum IgG PK parameters are presented for both dosing regimens in Table 4. The mean C_trough_ values (IgG levels collected before infusions on days 8 and 15 for weekly and biweekly dosing, respectively) were similar in the dosing regimens, whereas the C_max_ for the biweekly regimen was slightly higher than the C_max_ for the weekly regimen. As expected, the mean serum IgG AUC_0‐tau_ was approximately 2‐fold higher for the biweekly dosing regimen than the weekly dosing regimen (Table 4), as the mean given dose was also approximately 2‐fold higher for the biweekly treatment. The mean dAUC was similar for both biweekly and weekly dosing regimens (0.25 and 0.24 [g·h/L]/mg, respectively), as was the mean CL_ss_ (4.14 and 4.41 mL/h, respectively). Similarities between the dosing regimens were also observed for the mean C_trough_ and mean C_min_ (Table 4). Individual T_max_ varied significantly for both dosing regimens, from 0 to 5 days on the weekly dosing regimen and from 2 to 7 days on the biweekly dosing regimen. The median T_max_ for the biweekly dosing regimen was 3.02 days, and for the weekly dosing regimen it was 2.00 days. The individual patient serum IgG concentration‐time profile curves showed variability between patients (Figure 1). Median serum IgG concentrations are presented in Figure 2.

**Table 4:** Serum IgG PK Parameters by Treatment Regimen: Patients Who Completed Both PK Parts

PK Parameter, Unit	Summary Statistic	Weekly(n = 15)	Biweekly(n = 15)
AUC_0‐tau_, g·h/L^a^	Geometric mean (CV%) Mean (SD)	1681 (15.1) 1699 (256)	3562 (15.8) 3601 (531)
dAUC, (g·h/L)/mg^a^	Geometric mean (CV%) Mean (SD)	0.24 (18.0) 0.24 (0.05)	0.25 (14.3) 0.25 (0.04)
C_max_, g/L	Geometric mean (CV%) Mean (SD)	10.79 (15.5) 10.91 (1.67)	11.82 (17.0) 11.97 (2.02)
T_max_, day	Median Min, Max	2.02 0, 5.1	3.02 2.0, 7.1
C_trough_, g/L	Geometric mean (CV%) Mean (SD)	10.08 (16.3) 10.21 (1.63)	9.96 (19.7) 10.13 (1.94)
C_min_, g/L	Geometric mean (CV%) Mean (SD)	9.43 (19.9) 9.60 (1.93)	9.69 (17.8) 9.83 (1.73)
CL_ss_, mL/h	Geometric mean (CV%) Mean (SD)	4.35 (18.5) 4.41 (0.77)	4.08 (17.2) 4.14 (0.73)

AUC_0‐tau_, area under the serum concentration‐time curve from time zero through tau (tau = 7 days for a weekly regimen and 14 days for a biweekly regimen); CL_ss_, clearance at steady state (calculated as actual dose/AUC_0‐tau_); C_max_, maximum IgG concentration in serum; C_min_, minimum IgG concentration in serum during dosing interval; C_trough_, trough IgG concentration in serum, collected before next infusion during a treatment regimen; CV, coefficient of variation; dAUC, dose‐adjusted AUC_0‐tau_ (calculated as AUC_0‐tau_/actual dose); IgG, immunoglobulin G; PK, pharmacokinetic; SD, standard deviation; T_max_, time to reach C_max_.

a Note: N = 13: Two patients (0016 and 0025) were excluded from this analysis as an IgPro20 dose was administered within 1 or 2 days before the last PK sample on day 15 or day 8, respectively.

A double‐peak serum IgG concentration‐time curve with changes in serum IgG concentrations greater than 5% was observed in 5 weekly and 3 biweekly profiles, some of which are presented in Figure 1. A threshold of 5% was conservatively selected as a true change indicator based on the test‐retest reproducibility of the IgG assessment method. A dip in serum IgG levels occurred on Day 4 in most cases for weekly IgPro20 administration and on Day 6 in all cases for biweekly IgPro20 administration. In weekly administration profiles, the serum IgG concentration decreased by 0.5–4.1 g/L (corresponding to a relative decrease of 5.2%–35.0%), and then increased by 0.6–3.9 g/L (corresponding to a relative increase of 7.7%–51.3%) at the next oint collected. In biweekly profiles, there was an initial decrease of 0.7–1.6 g/L (corresponding to a relative decrease of 6.2%–14.5%), followed by an increase of 1.0–1.2 g/L (corresponding to a relative increase of 8.7%–12.7%) at the next measurement.

The ratios of biweekly to weekly values of several PK parameters were close to 1: the GMR (90%CI) of biweekly to weekly dAUC was 1.07 (1.03–1.10), and those of biweekly to weekly C_max_ and C_trough_ were 1.10 (1.06–1.13) and 0.98 (0.95–1.01), respectively.

## Discussion

The serum IgG exposures were equivalent with SCIG IgPro20 weekly and biweekly dosing regimens, with C_trough_ levels maintained within the normal range (∼10 g/L) that were slightly higher for weekly than biweekly dosing. Because the biweekly dose was equal to twice the individual weekly dose, peak serum IgG was expectedly higher for the biweekly dosing than weekly dosing regimen. Furthermore, in line with the previous reports, the median T_max_ for weekly dosing was 2 days and 3 days for biweekly dosing in the present study.^9^, ^10^ Of note, T_max_ was highly variable, ranging from 0 to 5 days with weekly dosing and from 2 to 7 days with biweekly dosing. This suggests that it is not always possible to predict the time of peak serum IgG concentration of SCIG products based on mean data. In fact, several study patients with double‐peak PK curves had the lowest concentrations of IgG during the assumed average T_max_ timepoints. The exact mechanism leading to high individual variability of PK profiles with both weekly and biweekly dosing regimens (Figures 1 and 2) is unclear. It likely reflects the complex interactions between the processes of slow IgG absorption from the subcutaneous depot via the lymphatic system, redistribution, and catabolism running in parallel, which results in poor predictability of T_max_ and in the interesting phenomenon of C_min_ occurring not necessarily at the C_trough_ time. This observation deserves further investigation, as it may provide insight into the PK processes of IgG in patients with PID, as well as for discussing its potential impact on the clinical practice of IgG replacement therapy.

When comparing the PK parameters (AUC, C_max_, and C_trough_) across dosing regimens, the GMR for each of the parameters was close to 1, and although not an objective of the study, these results indicate that the 90%CIs fell within the typical equivalence criteria of 0.80–1.25. These results provide evidence that for both weekly and biweekly dosing (at an equivalent total dose over 2 weeks), the observed IgG PK parameters demonstrated equivalent exposures. The PK characteristics of weekly infusion of SCIG have been described in clinical studies for various SCIG formulations^3^, ^26^ and also for IgPro20.^6^, ^24^ Gustafson and colleagues reported PK parameters of biweekly administration of a 16% SCIG for a total of 24 weeks in 12 patients,^19^ demonstrating that biweekly SCIG therapy results in high and stable serum IgG levels, offering an alternative therapy regimen to patients with PID. A recently published study provided evidence of the efficacy of the biweekly IgPro20 regimen in patients with PID.^27^ Similar results were obtained in our study, indicating that biweekly IgPro20 infusions in patients with PID maintained stable serum IgG levels.

Interestingly, double‐peak PK curves were observed in approximately half the patients with full PK profiles investigated in our study. In 8 of 17 patients, the rapid decrease/increase in serum IgG concentrations between 2 consecutive measurements was greater than 5% and in 3 patients greater than 10%. A similar double‐peak phenomenon was also reported in a previous study conducted in patients with PID treated with IgPro20 and was suggested to reflect IgG absorption kinetics in tissue.^6^ Indeed, IgG absorption may be a rate‐limiting step for release of the SCIG into the bloodstream.^6^ Although the reasons for this phenomenon are unclear, it may make the correct timing of the lowest‐level assessment to critically evaluate treatment adequacy based on target trough levels in SCIG‐treated patients more important than previously thought.

In line with the findings of this study, a recently published study provides further support for the efficacy of the biweekly IgPro20 regimen in patients with PID.^18^ Another recent study performed in patients with PID receiving 20% IgG demonstrated equivalent IgG serum exposure in daily and biweekly administration.^28^ In summary, biweekly SCIG infusion is a relatively new strategy that will increase flexibility for the treatment of PID and will provide more choices for both patients and clinicians.

## Conflicts of Interest

M.A.R., G.B., M.P., M.A.T., Y.Z., and J.H. are employees of CSL Behring. M.A.R., G.B., Y.Z., J.H., and M.A.T. own CSL Behring shares. H.C. has previously been a member of advisory boards for CSL Behring. E.H. is a consultant for Leadiant and receives investigator‐initiated grants from CSL Behring. Editorial and graphical support was provided by Vibhuti Singh, PhD, and Heather Shawcross, PhD, of Fishawack Communications GmbH, Basel, Switzerland, a member of the Fishawack Group of Companies, supported by CSL Behring.

## Funding

The data summarized in this study are from CSL Behring‐sponsored clinical trials.
